# MSCs and MSC-Exos in pediatric ARDS treatment: translational research and the reshaping of nursing roles

**DOI:** 10.3389/fped.2026.1850469

**Published:** 2026-07-17

**Authors:** Li Zhang, Lingli Jian

**Affiliations:** Department of Pediatrics, Daping Hospital, Army Medical University, Chongqing, China

**Keywords:** MSCs, MSC-Exos nursing role, pediatric ARDS, translational medicine

## Abstract

Pediatric Acute Respiratory Distress Syndrome (ARDS) is a severe lung condition with high mortality and limited conventional treatments. Mesenchymal stem cells (MSCs), which are special cells that can develop into a variety of cell types, and their tiny vesicles called exosomes (MSC-Exos), are highly promising cell-based therapies for pediatric ARDS because of their anti-inflammatory, immunomodulatory, and tissue-repair functions. This review summarizes current knowledge on MSCs and MSC-Exos in ARDS, including recent progress in studies before human trials and in actual patient trials. It focuses on challenges, needed competency changes, and how nursing roles are evolving in this new therapeutic field. The discussion provides a theoretical foundation for developing a nursing framework to support and enhance patient care with advanced cell therapies.

## 1 Introduction

Despite major advances in our understanding of pediatric ARDS, it remains one of the most frustrating conditions we face in critical care medicine. Sepsis, a common and often fatal condition in the pediatric intensive care unit (PICU). The standard-of-care management of these patients is still largely supportive, consisting mainly of lung-protective ventilation strategies, prone positioning, fluid restriction, or conservative fluid management; all of which may alleviate the condition but do not directly target molecular and cellular dysregulations that underlie this syndrome. However, the limitations of these contemporary methods have spurred vigorous exploration of new therapeutic measures that may modulate the immune response and enhance tissue repair, potentially targeting mechanisms underlying pediatric ARDS rather than just alleviating its symptoms.

Novel therapies, particularly cell-based and cell-derived therapeutics, have emerged in recent years as promising candidates that could revolutionize the treatment of pediatric ARDS, with MSCs and MSC-Exos being the most translatable frontiers. MSCs are a class of multipotent stromal cells found in multiple sources, including bone marrow, adipose tissue, and umbilical cord, with unique immunomodulatory, anti-inflammatory, and regenerative properties that make them an ideal candidate for targeting the complex pathogenesis of pediatric ARDS. This paradigm shift from cell replacement to the delivery of bioactive molecules as a therapeutic strategy has spurred even greater interest in MSC-Exos—nanoscale extracellular vesicles, such as exosomes, that are packed with proteins, lipids, and nucleic acids and mediate many of the beneficial effects imparted by their parent cells.

However, this translational journey is fraught with complexities, including optimizing cell sourcing and exosome isolation protocols, determining optimal dosing and timing regimens, and navigating regulatory hurdles for biologic products. In addition, their unprecedented nature necessitates a rethinking of well-established paradigms in clinical care, especially regarding the roles and responsibilities of everyone involved in the delivery and monitoring of these treatments. As one of the pillars of patient-centered care, nurses bring unique expertise that drives this transformation forward, requiring not only adaptation to new skill sets but also deeper interprofessional engagement to guarantee safe and effective integration of these novel interventions into clinical practice.

Consequently, the application of MSC and MSC-Exos therapies in pediatric ARDS management promises to take nursing roles to another level, requiring nurses to transition from simple executors of physicians' orders and traditional symptom monitoring to advanced process managers for biotherapeutic modalities. In particular, this extended scope includes a full grasp of cellular and exosomal biology, competence with the processing and delivery of live biological products, alert recognition of idiosyncratic effects such as infusion reactions or lympho-immune dysregulation that could arise during the course of therapy, and holistic education/psychosocial support for patients/families in experimental treatment situations. Given the extent of knowledge required to deliver these advanced therapies safely to patients, implementation and integration into clinical practice will hinge on a ready nursing workforce that can translate novel science into meaningful interventional benefit at the bedside while ensuring therapeutic impact across all settings without sacrificing patient safety or quality.

Therefore, systematically synthesizing the scientific foundations and current translational status of MSCs and MSC-Exos for pediatric ARDS, alongside proactively reviewing the nursing landscape, is key to guiding the safe, effective, and standardized progress of this field. This review provides a broad overview, focusing on mechanistic insights, clinical advances, and the critical role of nursing in integrating these therapies into intensive care practice, thereby directly impacting patient outcomes for those with this devastating syndrome.

### 1.1 Core therapeutic mechanisms: From direct cellular action to paracrine effects

**Figure 1:**
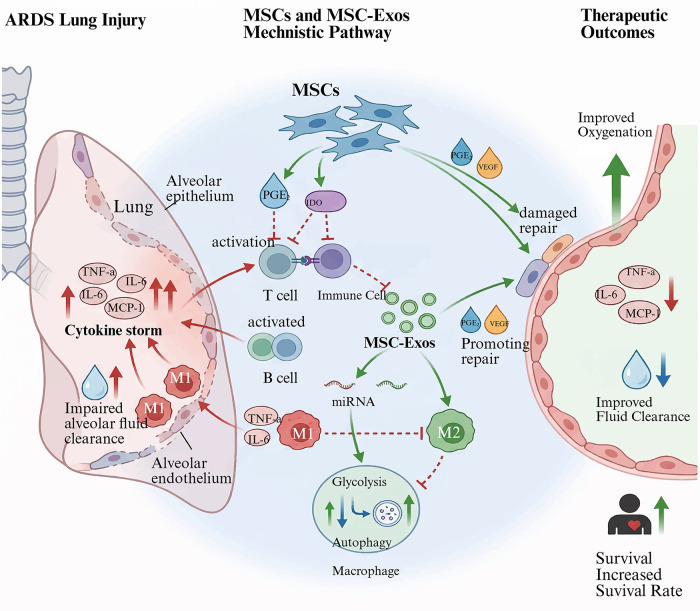
Core therapeutic mechanisms of MSCs and MSC-exos in pediatric ARDS. MSCs and MSC-Exos alleviate pediatric ARDS by suppressing the cytokine storm, shifting macrophages from M1 to M2 phenotype, and promoting alveolar epithelial/endothelial repair. These effects are mediated by soluble factors (PGE2, IDO), regulatory miRNAs (miR-377-3p), and growth factors (1–4). Figure created with BioRender.com and obtained relevant publishing licenses.

MSCs exert therapeutic effects in pediatric ARDS primarily through immunomodulation and paracrine signaling, rather than direct engraftment. Key mechanisms include suppression of the cytokine storm (via PGE2 and IDO), polarization of macrophages from pro-inflammatory M1 to reparative M2 phenotypes, and promotion of alveolar epithelial/endothelial repair (Figure 1) (1–4, 7, 8, 21).

In this paracrine arsenal, extracellular vesicles, especially exosomes, are pivotal effectors. These nanoscale vesicles transport mRNA, miRNA, and proteins to target cells in the injured lung (5–7). MSCs and their exosomes also enhance repair of the injured alveolar-capillary barrier. They deliver (8) growth factors, including keratinocyte and vascular endothelial growth factors, to promote alveolar epithelial and endothelial repair. This stimulates proliferation and differentiation of type II alveolar epithelial cells for lung reepithelialization and continued pulmonary surfactant production, and strengthens endothelial intercellular junctions to limit vascular leakage and pulmonary edema (9, 10). Evidence shows that bone marrow-derived stem cells (MSCs) can inhibit apoptosis of alveolar epithelial cells in ARDS via the CXCR4/CXCL12 axis, suggesting a targeted reparative mechanism (11). Thus, the primary therapeutic function of MSCs in pediatric ARDS is a multifactorial process comprising inhibition of immune dysregulation and stimulation of tissue repair, largely via their dynamic paracrine secretome and vesicular signaling.

### 1.2 Therapeutic advantages of MSC-Exos compared to MSCs

**Figure 2:**
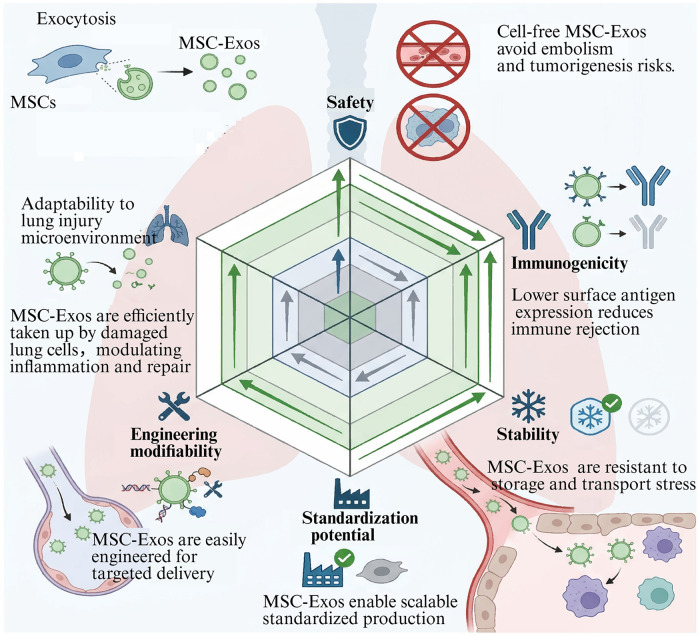
Comparative advantages of MSC-exos over MSCs. MSC-Exos offer lower immunogenicity, no risk of embolization or tumorigenicity, better stability, and greater potential for engineering (5, 6, 12, 13, 15, 17). Figure created with BioRender.com and obtained relevant publishing licenses.

MSC-Exos offer several advantages over whole MSCs, including lower immunogenicity, no risk of embolism or tumorigenicity, enhanced stability, and greater potential for engineering (Figure 2) (5, 6, 12, 15). These features position exosomes as a promising cell-free alternative, though standardization challenges remain (Section 2.3).

MSC-Exos have enhanced physicochemical stability and handling properties. In addition to shelf-life considerations, live MSCs are more demanding and often cryogenically preserved (13, 14), while exosomes can better withstand standardization for production, storage, and transport. Besides, MSC-Exos provide a more specific and customizable mode of action. Exosomes are the main effectors of MSC paracrine activity as they contain a defined molecular cargo that can be analyzed, characterized, and even engineered (6). This potential paves the way for “designer exosomes,” which modify vesicle content to amplify specific therapeutic functions, such as enhancing anti-inflammatory miRNA payload or adding targeting ligands for “targeted delivery” to traumatized lung tissue (15). This level of precision enables an “on demand design” strategy applicable to personalized medicine, which could circumvent the inherent heterogeneity across different MSC sources and preparations (16). Moreover, the cell-free nature of exosome therapy bypasses problems related to variability in MSC potency and the pernicious effects of the pro-inflammatory ARDS lung proteome on the viability and function of administered cells—a difficulty observed in several clinical studies (17, 18) (Figure 1).

2

### 2.1 Solid evidence and model advancements in preclinical research

Preclinical studies in LPS-, acid-, or cecal ligation-induced ARDS models have consistently shown that both MSCs and MSC-Exos improve oxygenation, reduce pulmonary edema, alleviate histopathological injury, and increase survival (19, 20). Mechanistic advances have identified key miRNAs and pathways (autophagy, glycolysis modulation) that mediate these effects (7, 21). Optimization studies suggest early intervention and combined intravenous/inhalation routes may enhance efficacy (22).

Beneficial effects of MSC-Exos in lung injury have been attributed to delivery of miR-377-3p, resulting in protective autophagyor (21). Studies are defining the optimal treatment window, the routes of administration, and the effective doses. There have been reports of combining inhalation and intravenous injections for systemically administering umbilical cord MSC-Exos, as a biophysical modus operandi to improve therapeutic effect, considering the dual-route administration provides two bioavailable sources in short order; local rapid relief would obtain first through the inhaled approach, followed by any remaining systemic efficacy occurring via an intravenous route (22).

In addition, the source and preconditioning of MSC-Exos are currently being addressed. Comparative analysis shows that MSCs from distinct tissues, including umbilical cord (UC-MSCs), bone marrow (BM-MSCs), and adipose tissue (AD-MSCs), have unique profiles associated with varying efficacy (Figure 2) (23). Pre-conditioning approaches, in which MSCs are pre-treated with hypoxia or inflammatory cytokines, have been used to promote a more beneficial immunomodulatory and reparative secretome that confers greater therapeutic potency (24). These extensive preclinical studies lay the groundwork for clinical translation, but also underscore the complexity of variables—source, preparation, and administration protocol—that should be standardized as much as possible prior to use in humans (Figure 2).

### 2.2 Challenges and preliminary results of clinical translational trials

MSCs and MSC-Exos have demonstrated preclinical efficacy against pediatric ARDS, but their ability to translate into practice and remedy remains highly uncertain, with early-phase trials beginning the long process of testing the waters and setting precedent despite yielding results that are only broadly informative. Phase I/II early clinical trials mainly evaluate safety and feasibility. To date, all such trials have been conducted in adult ARDS populations; no pediatric-specific trial has been published. Nevertheless, these adult studies demonstrate that intravenous allogeneic MSC infusions are generally safe and well tolerated (19). The favorable safety profile has been supported by meta-analyses of RCTs that found no significant increase in adverse events compared with many standard care controls (25). On the efficacy side, these early trials have sought potential biological signals. Some studies have shown reassuring trends, especially regarding COVID-19-related ARDS: improvement in the oxygenation index (PaO2/FiO2), decreases in systemic inflammatory biomarkers, better imaging recovery, and signs of decreased mortality (26–28).

The pilot study of human umbilical cord MSC-derived exosomes (hUCMSC-Exos) in patients with COVID-19 ARDS showed decreased levels of inflammation markers, including TNF-α and CRP, as well as regulation of immune cell subtypes (19, 20). Likewise, a meta-analysis suggested that MSC-based therapy may be associated with reduced mortality in ARDS (25). However, these signals of efficacy are exploratory and uncertain. Well-designed, rigorous Phase III RCTs that can provide consistent and definitive evidence of clinical benefit—e.g., a statistically significant reduction in hard endpoints like 28-day mortality, ventilator-free days, or duration of mechanical ventilation—are still missing (18).

A systematic review and meta-analysis of RCTs (all involving adult ARDS patients) found that no obvious improvement in other key outcomes such as hospital stay or 6-minute walk distance after treatment could be noted (18). Multiple powerful translational bottlenecks limit their widespread clinical adoption. A significant concern is the lack of standardized guidelines for cell and exosome production, characterization, and quality control, which results in product heterogeneity (19). Identifying the right patient population is complicated by the reuse of clinical and biological heterogeneity itself in ARDS; not all patients will respond, and there are currently no available predictive biomarkers to facilitate positive selection 30. Another significant obstacle to access is the cost associated with producing and administering these advanced therapeutics (19). Moreover, the extreme ARDS lung microenvironment may also impact MSC function and survival (17), further influencing their reparative potential. Solving these challenges — via standardized manufacturing, coordinated biomarker-driven patient stratification, complex system studies including dosage and administration optimization, and better appreciation of the underlying interactions between host and environmental factors— is paramount to translating MSC and MSC-Exos therapies from a potentially valuable experimental intervention into standard clinical practice for ARDS (24).

It is important to note that most available clinical evidence comes from adult ARDS studies, including those related to COVID-19. To date, no published pediatric trials of MSCs or MSC-Exos for ARDS exist, highlighting a critical knowledge gap and the need for caution when extrapolating adult data to children (Table 1).

**Table 1:** Key clinical studies of MSCs and MSC-Exos in ARDS.

Study/Reference	Population	Product Type	Source	Phase	Key Endpoints
START trial (19)	Adult ARDS	MSC	Allogeneic BM-MSC	I/II	Safety, oxygenation
REALIST trial (19)	Adult ARDS	MSC	Allogeneic BM-MSC	I/II	Safety, feasibility
Häberle et al. (26)	Adult COVID-19 ARDS	MSC	Not specified	Case series	Oxygenation, mortality
Hashemian et al. (27)	Adult COVID-19 ARDS	MSC	Perinatal tissue	Case series	Safety, clinical improvement
Hosseinzadeh et al. (29)	Adult COVID-19 ARDS	MSC-Exos	hUCMSC-Exos	RCT	Inflammatory markers, immune modulation
Kakabadze et al. (14)	Preclinical (rat)	MSC	Placenta-MSC	Animal study	Cytokines, tissue repair
Pediatric studies	None to date	–	–	–	No published pediatric trials.

Furthermore, while MSC-Exos have shown promise in preclinical models and a single adult trial (29), the majority of clinical safety and efficacy data still derive from whole MSCs rather than MSC-Exos, necessitating separate evaluation.

### 2.3 Standardization and heterogeneity challenges for MSC-exos

Despite the growing promise of MSC-Exos as a cell-free therapeutic strategy for pediatric ARDS, their clinical translation is hampered by considerable heterogeneity and lack of standardization across multiple domains. Table 2 summarizes the major sources of variability, including EV nomenclature, isolation and quality control (QC) methods, dosing metrics, and storage/handling protocols. These issues complicate cross-study comparison, hinder regulatory approval, and must be addressed through international consensus guidelines and good manufacturing practice (GMP) compliance before widespread clinical application (15). The challenges outlined here directly reinforce the translational bottlenecks discussed in Section 2.2.

**Table 2:** Key sources of heterogeneity and lack of standardization in MSC-Exo research.

**Category**	**Specific issues**	**Examples/consequences**	**References**
EV nomenclature	Interchangeable use of terms (exosomes, microvesicles, extracellular vesicles)	Difficulty comparing results across studies; imprecise definition of product	(5, 6)
Isolation methods	Ultracentrifugation, size-exclusion chromatography (SEC), polymer-based precipitation, immunoaffinity	Variable yield, purity, size distribution, and functional activity	(12, 15)
Quality control (QC) parameters	Particle concentration, marker proteins (CD9, CD63, CD81), endotoxin levels, purity ratio	Lack of unified thresholds; batch-to-batch variability	(6, 12)
Dosing metrics	Protein content (*μ*g), particle number (e.g., 1 × 10^9^), RNA quantity	Inability to directly compare efficacy across studies; unclear dose-response relationship	(12, 15)
Storage & handling	Temperature (−80 °C vs. −20 °C), freeze-thaw cycles, lyophilization, buffer composition	Altered exosome stability, aggregation, loss of bioactivity	(5, 6)

3

### 3.1 Special nursing challenges in the treatment implementation process

Nurses transition from task executors to advanced process managers, research coordinators, and patient advocates. Expanded competencies include cell therapy biology, infusion reaction monitoring, sample handling, ECMO support, and clinical trial ethics (32–34, 36, 42, 45, 47). Figure created with BioRender.com and obtained relevant publishing licenses.

**Figure 3:**
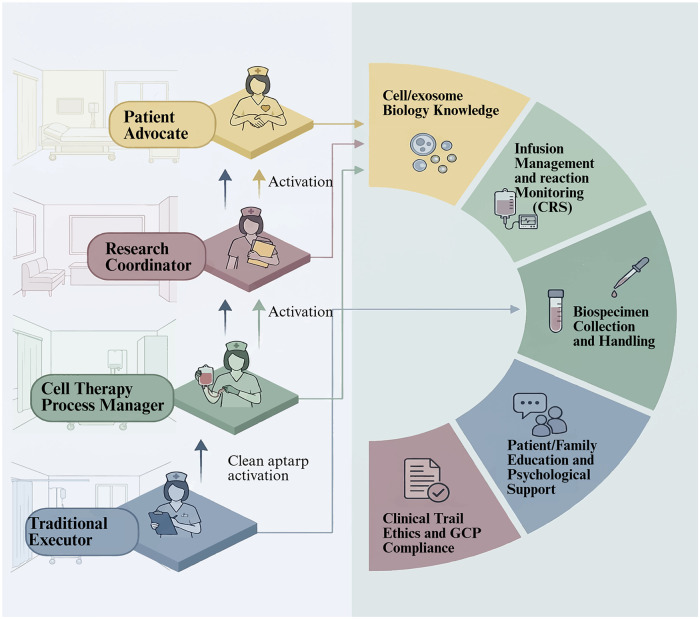
Evolution of nursing roles and core competency framework.

Administration of MSCS and MSC-Exos for pediatric ARDS presents several highly specialized nursing challenges that require meticulous procedural knowledge and vigilant monitoring. The emphasis on the complexity of infusion management and monitoring for these biologics raises a concern. MSCs and MSC-Exos differ from conventional pharmaceuticals in that they require specific handling protocols for thawing, reconstitution, and preparation, which are critical to ensure cell viability and therapeutic potency (8). Around, during, and after infusion, nurses must be ready to closely monitor for infusion-related reactions. Although the provided references did not point to individual adverse events for MSC/exosome infusions in pediatric ARDS, some comparative cell therapies like Chimeric Antigen Receptor (CAR) T–cell therapy have known risk profiles and also share a frequent phenomenon called cytokine release syndrome (CRS), characterized by fever and hemodynamic instability (33, 34) [ Figure 3].

Although the cytokine storm profile may differ, nursing staff must be acquainted with systemic inflammatory response profiles and recognize possible signs of allergic reactions and acute changes in cardiopulmonary baseline, particularly the reflex exacerbation of pulmonary edema (interstitial component) in patients already affected by pediatric ARDS. This requires proficiency in advanced hemodynamic monitoring and rescue protocols. Additionally, nurses are vital in recognizing and treating severe adverse reactions. Non-infectious inflammatory responses can be incited by cell-based therapies. Post-transplantation administering bendamustine, used for treatment in hematopoietic stem cell transplantation, has been associated with a very high incidence of CRS, characterized by elevated fever and liver function test abnormalities, as well as pancreatitis (35).

Likewise, MSC/exosome therapy may produce transient laboratory abnormalities or inflammatory symptoms that require expert differentiation from progression of underlying pediatric ARDS or a new-onset nosocomial infection. This can only be achieved through a nuanced understanding of the therapy and its mechanism, to avoid misattributing symptoms and ensure proper management. Ultimately, many MSC/exosome applications occur in research contexts, which necessitate stringent standards for the collection and management of biological samples. To assess therapeutic responses and mechanistic effects, such as modulation of macrophage polarization or reduction in inflammatory cytokines, tens to hundreds of µg (1–100 million cells) can be sampled at precise times from blood, bronchoalveolar lavage fluid (BALF), or other samples (22). Nurses are responsible for ensuring that these samples are obtained, processed, stored, and transported in accordance with the exact specifications of the clinical trial protocol. Any deviation can compromise the integrity of critical research data aimed at understanding treatment effects, such as changes in glycolysis in alveolar macrophages or levels of interleukins in BALF (22, 36). Thus, the nursing role expands to include precise research coordination at the bedside, directly impacting the quality and validity of translational science(Figure 3).

### 3.2 Expansion of the core nursing knowledge and skill system

The emergence of MSC and exosome therapies for pediatric ARDS will rapidly require a much broader core knowledge base and clinical skill set from critical care nurses, transitioning far beyond traditional supportive care to embrace both the concepts of regenerative medicine and complex clinical trial coordination. First, nurses need to master the basics of cell therapy. This includes knowledge of the basic biology of MSCs and MSC-Exos — their sources, main mechanisms of action, and potential adverse effects (8, 37). This knowledge is not academic; it is essential for conducting effective patient and family education, setting realistic expectations, and performing informed clinical surveillance. For example, understanding that exosomes may exert effects by inhibiting glycolysis in macrophages or delivering specific microRNAs allows nurses to better contextualize patient responses and laboratory findings (7, 21).

Concurrently, the severity of the patient population mandates proficiency in advanced life support and monitoring skills. Patients with pediatric ARDS severe enough to be candidates for experimental cell therapy are typically on advanced mechanical ventilation modes, require sophisticated hemodynamic monitoring, and may be supported by ECMO (38, 39). Nursing care for these patients is exceptionally complex, involving the management of ventilator settings to protect the lungs, meticulous titration of vasoactive drugs, and specialized, high-risk care associated with ECMO circuits, including monitoring for complications such as thrombosis or cannula-related issues (40, 41). The integration of a new biological therapy adds another layer of complexity to this already intensive care regimen. Perhaps most distinctively, in the clinical trial setting, the nursing role frequently expands to include research coordination and ethical practice capabilities. Nurses often function as crucial liaisons between the research team, the patient, and the clinical team. This involves ensuring the informed consent process is thorough, ethical, and comprehensible, particularly for complex therapies where therapeutic misconception is a risk (42, 43). They are responsible for maintaining strict adherence to protocol, from the timing of interventions to the precise documentation of adverse events. This requires an understanding of Good Clinical Practice (GCP) and the ethical frameworks governing experimental treatments, including issues of equity in access and the management of patient data (44). The nurse's role in safeguarding patient rights and welfare while facilitating rigorous scientific inquiry becomes a central pillar of the care-delivery model in the cell-therapy era for ARDS.

### 3.3 Practical implementation toolbox for PICU nurses

To translate the above competency framework into daily PICU practice and clinical trial settings, we provide below a practical implementation toolbox. These checklists and protocols are directly derived from the nursing challenges (Section 3.1) and expanded competencies (Section 3.2) described above. They are intended as a starting point for institutional protocol development and bedside nursing education (Table 3).

**Table 3:** Practical implementation toolbox for PICU nurses in MSC/exosome therapy.

Step	Category	Key Actions
1	Product receipt & handling	Verify identity & expiration Thaw/reconstitute per SOP Confirm viability (if cells) Document lot & preparation time
2	Pre-infusion assessment	Baseline vital signs (HR, BP, RR, SpO_2_) Respiratory status (vent settings, FiO₂, P/F ratio) Allergy & prior infusion reactions Current meds (immunosuppressants, anticoagulants)
3	Intra-infusion monitoring	Vital signs: q15 min × 1 h, then q30min Watch for CRS signs (fever, tachycardia, hypotension, hypoxia) Monitor for acute respiratory deterioration Emergency cart & team notified prior
4	Post-infusion & AE escalation	Vital signs: q1 h × 4 h, then per PICU protocol Lab monitoring (CBC, CRP, IL-6, LFTs, renal) Delayed reaction watch (24–48 h) Escalation: nurse → attending → PI
5	Family education	Simple explanation: “cells that calm lung inflammation” Set realistic expectations (experimental) Discuss possible side effects (fever, BP changes) Provide psychosocial support
6	Research sample & protocol	Coordinate blood/BALF collection timing Proper tubes & processing (−80 °C freezing Complete CRFs within 24h Report protocol deviations to coordinator

### 3.4 Draft core provisions of a nursing regulation for MSC/exosome therapy in pediatric ARDS

To operationalize the nursing framework described above, we present a set of illustrative draft core provisions for a nursing regulation specific to MSC-Exos therapy in pediatric ARDS. These provisions cover key steps from patient eligibility to post-infusion monitoring, research compliance, and nurse certification, and are intended for local adaptation by PICU institutions (Table 4).

**Table 4:** Draft core provisions of a nursing regulation for MSC/exosome therapy in pediatric ARDS.

**Items**	**Title**	**Key Actions**
1	Indications and eligibility verification	Verify patient meets all inclusion criteria per clinical trial protocol before product preparation
2	Product handling and quality check	Confirm label integrity, expiration date, no visible abnormalities; follow SOP for thawing/reconstitution
3	Pre-infusion preparation	Document baseline vital signs, respiratory status, allergy history; ensure emergency equipment and resuscitation team on standby
4	Intra-infusion monitoring	Record vital signs q15 min × 1 h then q30 min; immediately report signs of CRS (fever, hypotension, hypoxia) to attending physician
5	Post-infusion and adverse event management	Monitor ≥4 h post-infusion, observe for delayed reactions up to 48 h; activate escalation pathway (nurse → attending → PI) for grade ≥2 adverse events
6	Research sample collection and documentation	Collect blood/BALF at protocol-specified time points; process per GCP standards; document within 24h
7	Patient and family education	Provide age-appropriate information on experimental nature, side effects, and psychosocial support resources
8	Competency requirement	Only nurses who have completed a certified training program in cell therapy administration are authorized

4

### 4.1 From traditional implementer to collaborative treatment partner and patient advocate

The incorporation of MSCs and MSC-Exos into the treatment armamentarium for pediatric ARDS requires a pivotal transformation of the nursing role from a conventional content-oriented implementer to an integral team member and patient advocate within a multidisciplinary team (MDT). This transition is crucial given the complexity, novelty, and risk of cell-based therapies. The nurse, as a core member of the MDT comprising intensivists, pulmonologists, cell biologists, and pharmacists, provides invaluable input on nursing practice points during treatment planning and adjustment. This collaborative role is exemplified in the management of severe cases, such as those requiring ECMO support alongside MSC therapy, where nurses coordinate complex care logistics, monitor for synergistic or adverse interactions, and ensure seamless integration of both advanced life support and regenerative treatment modalities (45). The nurse's frontline position enables real-time assessment of patient responses, informing MDT decisions on treatment escalation, repeating MSC doses, or managing complications, thereby directly influencing therapeutic trajectories and safety outcomes (26). Beyond the MDT, the nurse assumes the pivotal role of patient advocate and educator, tasked with demystifying this novel therapy for patients and their families. This involves explaining the biological rationale of MSCs—such as their immunomodulatory, anti-inflammatory, and tissue-reparative functions—while managing expectations by transparently discussing the current evidence, including promising early results on pulmonary function improvement and the variability in mortality reduction observed in clinical trials (1, 46).

Children with ARDS, especially in the context of severe conditions such as COVID-19, suffer from profound distress and anxiety (47); therefore, it is important to offer continued psychosocial support during treatment. The nurse champions the patient and family concerns in the care discussion. In addition, the nurse becomes the primary custodian of patient safety and the quality of treatment. This includes creating, implementing, and auditing standardized nursing operating procedures (SOPs) for cell therapy that span the entire process from receipt and handling of cellular products to administration of infusion and post-infusion monitoring (27). Creating comprehensive checklists for monitoring adverse reactions and relevant emergency measures is crucial for the timely diagnosis and treatment of possible infusion-related or delayed complications (1). Through the careful charting of physiological parameters, biomarker trends, physical findings, and clinical symptoms, nurses generate high-fidelity data fundamental to both assessing therapeutic efficacy and describing adverse safety outcomes- all while taking center stage in protecting the patient and informing the growing body of collaborative clinical evidence for such novel interventions (26, 28).

### 4.2 Building a specialized training and certification system for cell therapy nurse

**Figure 4:**
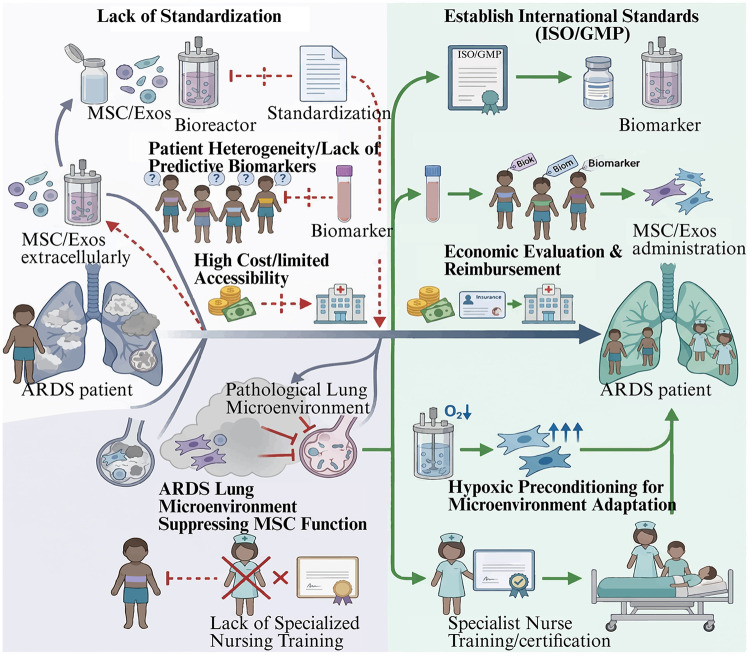
Translational challenges and solution pathways. Major challenges include lack of standardization, patient heterogeneity, high costs, and limited nursing training. Solutions involve international manufacturing standards, biomarker-driven personalization, health economic evaluation, and specialized nurse certification programs. (17, 19, 24, 30, 31, 49, 50). Figure created with BioRender.com and obtained relevant publishing licenses.

An appropriately trained and competent nursing workforce is essential to ensuring the safe and effective translation of MSC-based therapies for pediatric ARDS at scale; this requires the development of a standardized framework rooted in nursing education and professional development that goes beyond generalist critical care competencies towards specialization in cell therapy as a therapeutic class. A major foundation of this system is a comprehensive, standardized training curriculum. These programs need to impart fundamental knowledge about the biology of cell therapy, including MSC sources, mechanisms of action, and the pathophysiology specific to pediatric ARDS (4) [Figure 4].

This theoretical basis needs to be combined with high-level critical care skills applicable to the pediatric ARDS cohort, including advanced ventilatory strategies, hemodynamic monitoring, and management of patients on adjuvant support, such as ECMO (45). A special emphasis here must be placed on the ethical aspects of clinical research and obtaining informed consent in a setting where complex experimental therapies are used, such that there will need to be rigorous training in clinical research ethics, GCP, as well as advanced techniques for soliciting informed consent from patients with severe diseases (48). The modality of the training model needs a combination of didactic teaching, high-fidelity simulation for scenarios such as anaphylaxis or acute clinical deterioration after infusion, and clinical practicums under supervision at designated treatment centers (27). In addition, formal initial and ongoing recognition of advanced practice roles such as “Cell Therapy Clinical Nurse Specialist” or “Critical Care Cell Therapy Nurse” is required to institutionalize this expertise. These specialists would become the unit's experts, providing stakeholders with up-to-date knowledge, leading direct quality improvement efforts to optimize SOPs, and managing complex cases alongside mentoring general navigation staff (49). They help create uniformity in both the image and the quality of care across all areas of the health care system. Finally, the closure of the professionalization pathway must come from inspiring nursing leaders to engage fully with the scientific and guideline development underpinning their respective fields. Drawing on this experiential knowledge, nurse-leaders should contribute to collaborative writing of evidence-based clinical practice guidelines for the nursing care of individuals receiving MSC therapy as a treatment option for ARDS (50).

Furthermore, they should be empowered to initiate and lead nursing-focused clinical research. Such studies could investigate the impact of specific nursing interventions—such as structured pre-infusion education protocols on patient anxiety, or specialized mobilization strategies post-therapy—on patient-centered outcomes, including quality of life, psychological well-being, and long-term recovery metrics (51). This triad of standardized education, formalized advanced roles, and active participation in research and guideline development is essential for building a sustainable, competent, and influential nursing workforce capable of supporting the translation and optimization of stem cell therapies in critical care (Figure 4).

### 4.3 Example of draft core provisions for a nursing regulation on MSC-Exos therapy

To provide a concrete example of how such a regulation might be structured, we propose below a set of draft core provisions. These provisions are illustrative and intended for institutional adaptation, which is manifested in Section 3.4. They cover the key steps of MSC/Exosome therapy from patient eligibility to post-infusion follow-up, research compliance, and nurse certification (Table 4).

### 4.4 Vision for enhancing nursing competencies in cell and cell-free therapy for ARDS

To translate MSC and exosome therapies into safe and effective pediatric ARDS care, we propose the following strategic directions for nursing competency development:

1. Tiered training pathways—Establish foundational, advanced, and specialist-level training programs in cell therapy administration, aligned with the competency framework.
2. Formal certification—Develop national or regional certifications that require both theoretical examination and supervised clinical practice.
3. Multidisciplinary integration—Position nurses as equal partners in MDT rounds, protocol development, and adverse event committees, moving beyond the traditional executor role.
4. Nursing-led research—Empower nurses to lead implementation science projects, quality improvement initiatives, and patient-reported outcome studies.
5. Ethical and regulatory training—Mandate education on informed consent, Good Clinical Practice (GCP), and ethical frameworks for pediatric cell therapy trials.

## 5 Conclusion

MSC therapy and MSC-Exos represent a promising multi-targeted approach for pediatric ARDS, offering immunomodulation, alveolar repair, and reduced pulmonary edema. Exosome-based therapies provide advantages in safety, stability, and controllability, making them attractive for clinical translation.

However, translational challenges remain, including standardization of cell or exosome sourcing, isolation, dosing, and the need for personalized strategies based on pediatric ARDS endotypes. Rigorous health economic analyses are also imperative.

The integration of these biotherapeutics necessitates a parallel evolution of nursing practice. Traditional task-based care must transition to an advanced, integrated model where nurses understand therapeutic mechanisms, monitor for adverse events, manage cell product logistics, and educate patients and families. At the clinical care level, there is no alternative to empowering the nursing workforce. This demands the implementation of structured, bespoke training pathways to develop expertise in cellular therapy management.

To prepare the nursing workforce for safe and effective implementation, we propose five strategic directions: Tiered training pathways; Formal certification; Multidisciplinary integration; Nursing-led implementation science. Ethical and regulatory literacy Achieving this vision requires collaborative efforts from educators, administrators, policymakers, and nurse leaders. Through interdisciplinary partnership and advanced nursing education, we can translate MSC and exosome therapies into safe, equitable, and compassionate care for children with ARDS.

MSC and exosome therapy represent a new frontier in the treatment of pediatric ARDS. Its development has demonstrated the necessity of balancing innovative therapeutic potential with robust scientific and practical scrutiny. The success of this innovation, however, will not be determined just by biological considerations, but also by our capacity to adapt the healthcare ecosystem around it. Through interdisciplinary partnership, advanced nursing education and role development, and a clear-eyed view of the dual challenges posed by the science at stake here as well as caring for patients who have it or those they love. The aim is to translate this exciting science into safe, effective, equitable, and compassionate care to benefit patients suffering from this devastating syndrome.
